# Development of Defect-Rich WO_3-x_/TiO_2_ Heterojunction Toward Dual-Functional Enhancement: Boosting SERS and Photocatalytic Performance

**DOI:** 10.3390/nano15070521

**Published:** 2025-03-30

**Authors:** Xunfei He, Yinyan Gong, Lengyuan Niu, Can Li

**Affiliations:** Institute of Optoelectronic Materials and Devices, College of Optical and Electronic Technology, China Jiliang University, Hangzhou 310020, China; hxf2487748@163.com (X.H.); niulengyuan@163.com (L.N.); canli1983@gmail.com (C.L.)

**Keywords:** surface-enhanced Raman scattering, photocatalysis, heterojunction, WO_3-x_, TiO_2_

## Abstract

Semiconductors have emerged as promising candidates for surface-enhanced Raman scattering (SERS) applications due to their inexpensiveness and good chemical stability. Nevertheless, their low enhancement ability compared to noble metals makes it desirable to explore strategies for improving SERS performance. Since charge transfer (CT) between semiconductors and analytes plays a crucial role on the chemical enhancement mechanism of SERS, heterojunction engineering, a powerful method to boost optoelectronic performance via tailoring interfacial charge transfer, provides a promising approach. Here, we prepared defect-rich WO_3-x_/TiO_2_ nanocomposites via a facile solvothermal method to achieve dual-functional enhancement in SERS and photocatalytic activity. Due to suppressed recombination of charge carriers in WO_3-x_/TiO_2_ heterojunction with type II band alignment, more photogenerated carriers are available for CT, consequently increasing molecular polarizability. The SERS intensity of WO_3-x_/TiO_2_ is at least three times that of its component semiconductors, with a detection limit of 10^−10^ M for methyl orange (MO). Meanwhile, the suppressed recombination of charge carriers also results in higher degradation efficiency of WO_3-x_/TiO_2_ heterojunction (93%) than WO_3-x_ (47%) and TiO_2_ (54%) under visible-light irradiation for 120 min. This work provides insightful information on the development of dual-functional semiconductor systems through band structure engineering for ultrasensitive sensing and efficient remediation of environmental pollutants.

## 1. Introduction

The growing concerns of aquatic environment pollution by contaminants in industrial and domestic sewage (e.g., pesticides, antibiotics, azo dyes) have attracted great interest in developing dual-functional materials capable of detecting toxic chemicals at trace level in water and decomposing them by utilizing solar energy. Owing to rapid response, molecular fingerprinting, and ultrahigh sensitivity, surface-enhanced Raman scattering (SERS) has emerged as a promising analytical technique for various applications, including biomedical diagnostics, environment monitoring, and homeland security [1,2,3,4]. The significant enhancement in Raman signals is mainly ascribed to two mechanisms: the electromagnetic enhancement mechanism (EM), originated from intensified electromagnetic fields in the vicinity of nanostructure surfaces induced by localized surface plasmon resonance (LSPR), and the chemical enhancement mechanism (CM), aroused from charge transfer (CT) between adsorbed analyte molecules and substrates, which results in an increase in molecular polarizability [5,6]. Noble metals (e.g., Au, Ag, Cu) are popular candidates for fabricating SERS sensing platforms due to their strong LSPR under laser excitation, commonly used in Raman spectrometers [7], and exceptional enhancement factors (EF, 10^7^–10^14^) have been achieved for rational designed nanostructures with controlled morphology and composition, such as Au dual-gap nanodumbbells [8], porous-spiny Au-Ag nanoparticles [9], Au nanoprisms [10], Au@Ag core–shell nanoislands [11], and Ag-Cu Alloy microflowers [12].

In recent years, semiconducting materials have emerged as promising candidates for fabricating SERS substrates, owing to their inexpensiveness, exceptional chemical stability, and good spectral repeatability [13,14,15,16]. Various research works have demonstrated the Raman enhancement capabilities of semiconductor-based substrates. For instance, Jing et al. fabricated defective Cu_2_O and achieved a remarkable low detection limit (LOD) of 0.3 mg/kg for Sudan red III in herbal medicine extracts [17]. He et al. developed SERS-active substrate using few-layered van der Waals MoO_3_ nanosheets with EF and LOD of 2.28 × 10^4^ and 2 × 10^−8^ M for rhodamine 6G, respectively [18]. Qiu et al. synthesized hollow CuS for detecting residual tumor lesions [19]. It is important to note that the CuS substrate is capable of self-clearance from tumor tissues under near-infrared laser irradiation due to photothermal effects, resulting in the disintegration of CuS shells into small particles. Pan et al. designed hydroxyl-functionalized colloidal TiO_2_ nanocrystals for SERS sensing of polymerization inhibitors, such as 4-tertbutylcatechol (4-TBC) and hydroquinone (HQ) [20]. Due to strong electronic coupling with the phenolic hydroxyl groups, the colloidal TiO_2_ substrates are able to directly detect 4-TBC and HQ in olefins at a concentration as low as 0.9 ppm. Despite these advances, the SERS enhancement ability of semiconductor substrates, which primarily relies on the CM mechanism, is typically several orders of magnitude lower than that of noble metal substrates, severely limiting their practical applications [5,21,22]. Hence, there is an urgent need for the development of strategies to improve the SERS activity of semiconductor-based substrates.

Considering the critical role of photoinduced charge transfer on the CM mechanism, various nanostructure synthesis and post-growth processing methods have been proposed to promote the separation and transfer of photogenerated electron–hole pairs. Consequently, more charge carriers are available for the CT process between analyte molecules and substrates, resulting in an increased molecular polarizability tensor and enhanced SERS signals. Incorporation of defects (impurity and vacancies) can promote the SERS activity of semiconductors by modulating the electronic band structure and creating impurity levels in forbidden gap. For instance, Liu et al. showed that yitterbium doping can improve the SERS activity of titanium dioxide and the intensity achieved from the optimal sample is approximately five times that of pristine TiO_2_. Based on the results of experimental and DFT simulation, Liu et al. proposed that the enhancement can be attributed to a narrower band gap, higher density of states near the Fermi level, and stronger electrostatic attract to analyte molecules [23]. Xu et al. reported that lithium doping can effectively increase the SERS performance of zirconia due to reduced band gap energy and improved charge transfer [24]. Cao et al. improved the SERS sensitivity of MoO_2_ by manipulating oxygen vacancy concentration through a lithium reduction method and the detection limit is lowered to 10^−7^ M [25]. Similarly, Sun et al. found that the SERS sensitivity of WO_3_ nanosheets can be improved by introducing oxygen defects through sodium borohydride reduction [26]. Quan et al. demonstrated that the intensity of the SERS signal was increased by two times through the cooperative regulation of oxygen defects and amorphous state in TiO_2_ [27].

Construction of semiconductor heterojunctions (homojunctions) with staggered band alignment is a frequently used method to improve photocatalytic performance by promoting the separation and transfer of photoexcited electron–hole pairs. Given more charge carriers are available for the CT process between adsorbed analyte molecules and semiconductor substrates, the development of type II heterojunction appears to be a promising strategy for enhancing SERS performance. For example, Jiang et al. found that the SERS signal can be increased by nine times via the construction of TiO_2_/ZnO heterojunction, which was attributed to suppressed recombination of photoinduced charge carriers and a promoted CT process [28]. Similarly, He et al. developed CeO_2_ homojunction by combining two crystal planes with different conduction and valence band positions and achieved significantly improved SERS activity [29]. Although previous studies have demonstrated the potential of improving SERS activity by fabricating semiconductor compounds with a defined electronic band structure, the fundamental mechanism is still not thoroughly understood. It is also desirable to conduct a systematic study of its effect on dual-functional enhancement in SERS and photocatalytic activity.

Tungsten trioxide is a typical transition metal oxide semiconductor with many polymorphs and nonstoichiometric compositions containing rich oxygen vacancies. WO_3-x_, with structural flexibility and tunable electronic and optical properties, shows great potential for various applications, including chromic display, gas sensing, photocatalysis, photothermal therapy, and SERS sensing [26,30,31,32,33,34]. Meanwhile, titanium dioxide has been frequently employed to fabricate heterojunction nanocomposites for many photoelectronic applications owing to its abundance, excellent chemical stability, non-toxicity and self-cleaning ability [32,35,36,37]. With staggered band alignment, WO_3-x_/TiO_2_ heterojunction appears to be an ideal candidate for investigating the construction of semiconductor/semiconductor heterojunction as an efficient and low-cost approach to boost SERS activity. In addition, rich oxygen vacancies in WO_3-x_ can give rise to defect levels in its forbidden gap, providing extra CT pathways. In this work, we prepared WO_3-x_/TiO_2_ heterojunction nanocomposites by a facile solvothermal method and conducted a systematic study on their SERS and photocatalytic activity using methyl orange (MO) as probe molecules. It was found that WO_3-x_/TiO_2_ heterojunction nanocomposites exhibit a clearly improved SERS performance and the SERS intensity of the optimized heterojunction sample is three times that of pristine WO_3-x_, while negligible signal was detected for pure TiO_2_. The enhancement factor and low detection limit are 1.2 × 10^5^ and 10^−10^ M, respectively. The improved SERS activity of WO_3-x_/TiO_2_ heterojunction is attributed to reduced recombination of photogenerated electrons and holes, as confirmed by electrochemical impedance spectroscopy measurements. Therefore, more charge carriers are available for CT process between substrates and analyte molecules, resulting in amplified molecular polarizability. Furthermore, WO_3-x_/TiO_2_ also shows superior photocatalytic activity to pure WO_3-x_ and TiO_2_ with good stability. Additionally, a WO_3-x_/TiO_2_ heterojunction sample was used as a SERS platform to monitor the photodegradation process and the result is in reasonable agreement with traditional UV-Vis spectroscopy. The findings of this work help to gain a better understanding of the effect of heterojunction engineering on dual-functional enhancement in SERS and photocatalytic performance, providing more possibilities for further designing multifunctional materials by utilizing the interior properties of semiconductors.

## 2. Materials and Methods

### 2.1. Synthesis of WO_3-x_/TiO_2_

Tungsten (VI) chloride (WCl_6_, ≥99%), ethanol (anhydrous, ≥99.5%), ethylene glycol (anhydrous, ≥99.8%), potassium hydroxide (≥85%) and Nafion perfluorinated resin solution (D521CS, polymer content: 5.0–5.4%) were purchased from aladdin (Shanghai, China). and methyl orange (MO, 85%) was purchased from Tianjing Fuchen Chemical Reagent Factory(Tianjing, China). Deionized water (18.2 MΩ·cm) was used in all experiments.

WO_3-x_/TiO_2_ heterojunction nanocomposites were synthesized by a facile solvothermal method [38]. WCl_6_ (0.8 g) was dissolved in 32 mL of a mixed solution of ethanol and ethylene glycol (*v*:*v* = 9:1) under magnetic stirring for 10 min. Subsequently, a given amount of commercial TiO_2_ (Degussa P25, ≥99.5%) was added into the obtained WCl_6_ solution, and the mixture was treated by ultrasonic treatment for 30 min and magnetic stirring for 30 min. Then, the obtained suspension was transferred into a 50 mL stainless-steel autoclave and reacted at 180 °C for 12 h. After being naturally cooled down, blue precipitates were collected by centrifugation, washed thoroughly by deionized water and ethanol, and dried at 60 °C. A series of WO_3-x_/TiO_2_ heterojunction nanocomposites were prepared by using different amounts of P25 and labeled as WT-1 (m_WCl_6__:m_TiO_2__ = 5:1), WT-2 (m_WCl_6__:m_TiO_2__ = 25:1), WT-3 (m_WCl_6__:m_TiO_2__ = 50:1), WT-4 (m_WCl_6__:m_TiO_2__ = 75:1), WT-5 (m_WCl_6__:m_TiO_2__ = 100:1), corresponding to a weight ratio of WCl_6_:TiO_2_ at 5:1, 25:1, 50:1, 75:1, 100:1, respectively. Pristine WO_3-x_ was synthesized by the same procedure without TiO_2_.

A detailed description of material characterization employed in the present study is provided in Section S1 of the Supplementary Materials.

### 2.2. SERS Performance

For SERS measurements, as-prepared WO_3-x_/TiO_2_ heterojunction substrates and pure WO_3-x_ and P25 were dispersed in deionized water by ultrasonic treatment (1 mg/mL), which were then mixed with MO solutions at a volume ratio of 1:1 under constant stirring in the dark for 30 min. Then, 50 µL of the obtained mixtures with MO concentration (5.0 × 10^−5^ M) was drop-cast evenly on silicon chips (1 cm × 1 cm) and dried. Unless otherwise specified, SERS spectra were recorded on a Renishaw Invia micro-Raman spectrometer equipped with 532 nm laser (laser power = 0.1 mW at sample surface) at room temperature. A microscope objective lens at 50× magnification (NA = 0.75) was used to collect the Raman signals. The exposure time was 3 s and accumulation was carried out 3 times. For each sample, SERS spectra were collected from multiple spots and the average spectra were used in the discussion section. To evaluate SERS stability, SERS spectra were collected from WO_3-x_/TiO_2_ heterojunctions subjected to storage of 15, 30, 60 and 100 days. To exam the low detection limit, MO concentrations in the mixture varied from 5.0 × 10^−5^ M to 2.5 × 10^−10^ M. As a reference, a normal Raman spectrum of MO was measured by drop-casting 50 μL of MO solution (1 × 10^−3^ M) on silicon wafer and dried. In addition, the SERS spectrum of MO (5.0 × 10^−5^ M) on WO_3-x_/TiO_2_ was measured under 785 nm laser excitation (laser power = 0.6 mW at sample surface), 12 s exposure time and 9 accumulations.

### 2.3. Photocatalytic Performance

Briefly, 30 mg of WO_3-x_/TiO_2_ heterojunction samples was dispersed in 50 mL of MO solutions (25 mg/L) in quartz tubes under constant stirring in the dark for 30 min to reach the adsorption–desorption equilibrium. After that, the suspensions were subjected to visible-light irradiation from a 400 W halogen lamp equipped with a long-pass cutoff filter (λ ≥ 400 nm). At given time intervals, 4 mL of the suspensions was withdrawn and filtered through a 0.22 μm membrane, and clear solution was collected for UV-Vis absorption spectra measurements. Photodegradation efficiency (C_t_/C_0_) is evaluated by the reduction in the MO absorbance peak according to Lambert–Beer Law, where C_t_ and C_0_ represent MO concentrations in filtrates collected at time t and at adsorption equilibrium, respectively. To examine reusability, WO_3-x_/TiO_2_ was collected after each run of photodegradation experiment, washed, dried, and reused in the next run of photocatalytic experiments.

In addition, SERS was also used to monitor the photocatalytic activity of WO_3-x_/TiO_2_. At given time intervals, 50 µL of the suspensions was withdrawn and drop-cast directly on Si wafers. SERS spectra were measured under the same conditions as those described in Section 2.2.

## 3. Results and Discussion

### 3.1. Characterization of WO_3-x_/TiO_2_

**Figure 1:**
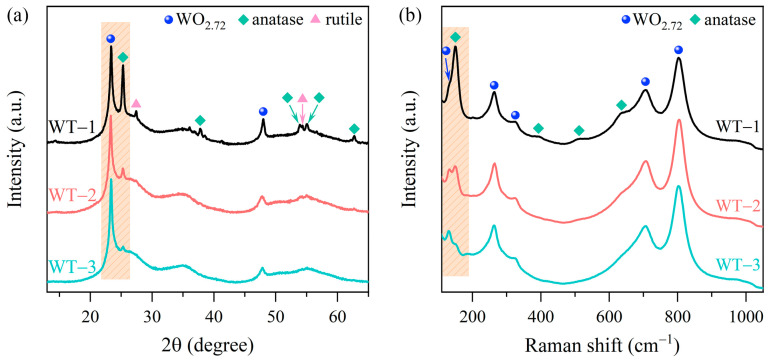
(**a**) XRD patterns of heterojunction samples WT-1, WT-2 and WT-3 prepared at the weight ratios of WCl_6_:TiO_2_ = 5:1, 25:1, 50:1, respectively. (**b**) Corresponding Raman spectra.

X-ray diffraction (XRD) curves of WO_3-x_/TiO_2_ heterojunction and pristine WO_3-x_ and TiO_2_ are shown in Figure 1a and Figure S1. It can be found that pure WO_3-x_ exhibits clear diffraction peaks which can be indexed to monoclinic WO_2.72_ (PDF card No. 73-2177), demonstrating the successful growth of tungsten trioxide with rich oxygen vacancies, as shown in Figure S1a. The XRD pattern of Degussa P25 reveals well-defined diffraction peaks originated from anatase phase (PDF card No. 21-1272) and rutile phase (PDF card No. 21-1276) of titanium dioxide (Figure S1b), with an estimated weight ratio of anatase/rutile = 80%:20% [39]. As depicted in Figure 1a, the XRD patterns of WO_3-x_/TiO_2_ heterojunction nanocomposites show clear reflection peaks originated from WO_2.72_ and commercial TiO_2_ without any detectable impurity diffraction features. For heterojunction nanocomposites WT-1, WT-2 and WT-3, the relative peak intensity of *I*_101_(anatase) to *I*_010_(WO_2.72_) is decreased and the (101) of TiO_2_ becomes barely detectable when the weight ratio of WCl_6_ to P25 is changed from 5:1 to 50:1, indicating a lower content of TiO_2_ in as-prepared WO_3-x_/TiO_2_ nanocomposites. Raman spectroscopy was employed to further characterize the microstructure of WO_3-x_/TiO_2_ heterojunction nanocomposites. Figure S1c shows the Raman spectrum of pristine WO_3-x_. The observed vibration modes at 131 cm^−1^ and 186 cm^−1^ can be attributed to lattice modes of monoclinic tungsten oxides [40], the Raman features at 263 cm^−1^ and 326 cm^−1^ are assigned to the O–W–O bending vibration modes [41,42], and the double peaks at 707 cm^−1^ and 804 cm^−1^ are ascribed to the stretching modes of O–W–O [41,42]. In addition, there is a very weak and broad band in the range of 960–1020 cm^−1^, which is related to the stretching vibration of terminal W=O mode [31,40]. As depicted in Figure S1d, the Raman spectrum of commercial TiO_2_ shows the characteristic Raman peaks originated from anatase TiO_2_ at 143 cm^−1^ (E_g_), 196 cm^−1^ (E_g_), 395 cm^−1^ (B_1g_), 516 cm^−1^ (A_1g_ + B_1g_) and 636 cm^−1^ (E_g_) [43]. The weak Raman band centered at 448 cm^−1^ (E_g_) in the inset of Figure S1d is related to rutile phase [44]. As shown in Figure 1b, the Raman spectra of WO_3-x_/TiO_2_ heterojunction nanocomposites reveal characteristic vibration features associated with both WO_2.72_ and TiO_2_. Similar to the tendency of XRD patterns, the intensity of TiO_2_-related peak is decreased for samples prepared at a higher weight ratio of WCl_6_ to P25. The results of XRD and Raman demonstrate successful synthesis of WO_3-x_/TiO_2_ heterojunction nanocomposites and the relative content of both components can be adjusted by the ratio of precursors.

**Figure 2:**
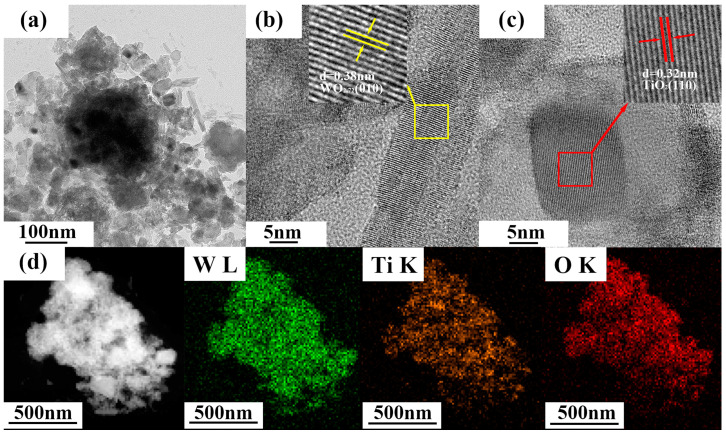
(**a**) TEM, (**b**,**c**) HRTEM and (**d**) EDS mapping images of WO_3-x_/TiO_2_.

The morphology of WO_3-x_/TiO_2_ heterojunction was investigated by transmission electron microscopy (TEM) and high-resolution TEM (HRTEM) observations. As shown in Figure 2a, the heterojunction sample WT-2 is composed of aggregations of poly-dispersed WO_3-x_ nanorods and TiO_2_ nanoplates. From Figure 2b,c, the HRTEM images reveal distinct lattice fringes with 0.38 nm and 0.32 nm interplanar spacings, corresponding to the (010) plane of WO_3-x_ and (110) plane of TiO_2_, respectively. In addition, elemental mapping images in Figure 2d show nearly uniform spatial distribution of Ti, W and O in the selected area, indicating that WO_3-x_/TiO_2_ heterojunction should be widely distributed in the obtained sample.

**Figure 3:**
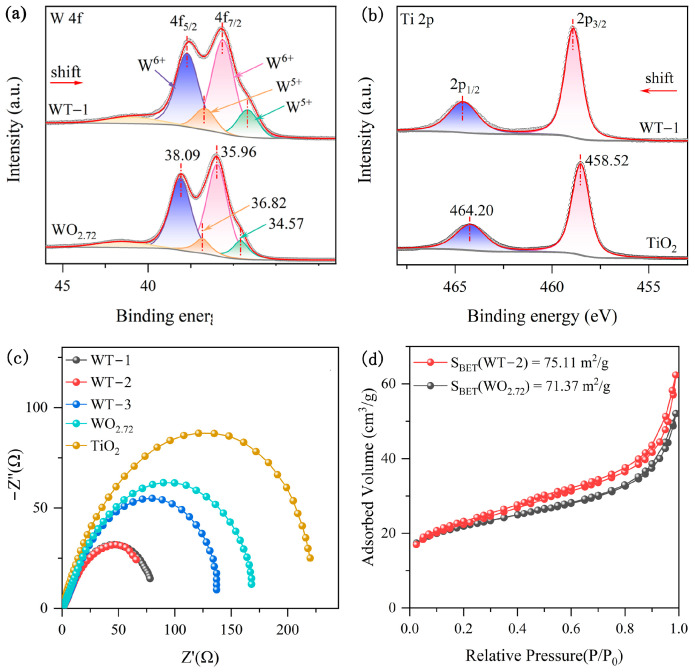
(**a**) W 4f XPS spectra of WO_3-x_/TiO_2_ and WO_3-x_. (**b**) Ti 2p 4f XPS spectra of WO_3-x_/TiO_2_ and TiO_2_. (**c**) Nyquist plot of WO_3-x_/TiO_2_ heterojunction samples and pure WO_3-x_ and TiO_2_. (**d**) N_2_ adsorption−desorption isotherms of WO_3-x_/TiO_2_ and WO_3-x_.

Moreover, we conducted X-ray photoelectron spectroscopy (XPS) measurements to analyze the chemical compositions and oxidation states of WO_3-x_/TiO_2_ heterojunction and pure WO_3-x_ and TiO_2_. As depicted in the survey spectra, pure WO_3-x_ shows distinct binding energy (BE) peaks of W 4f and O 1s (Figure S2a), while pristine TiO_2_ exhibits BE peaks of Ti 2p and O 1s (Figure S2b). Comparatively, WO_3-x_/TiO_2_ heterojunction reveals characteristic peaks originating from W 4f, Ti 2p and O 1s. Figure 3a displays the W 4f XPS spectra of WO_3-x_/TiO_2_ heterojunction and pristine WO_3-x_, exhibiting binding energy peaks associated with W 4f_7/2_ and 4f_5/2_. Moreover, the W 4f spectrum of WO_3-x_ can be fitted by two sets of doublet peaks, where the BE peaks at 35.96 eV and 38.09 eV can be attributed to W^6+^ 4f_7/2_ and 4f_5/2_, respectively, while the doublet peaks at 34.57 eV and 36.82 eV can be ascribed to W^5+^ 4f_7/2_ and 4f_5/2_, respectively [31,41,45], confirming the synthesis of non-stoichiometric tungsten trioxide with rich oxygen vacancies. The existence of rich oxygen vacancy defects was further confirmed by the existence of an O 1s peak associated with adsorbed oxygen species at the defect sites, as shown in Figure S3. Similarly, the W 4f spectrum of WO_3-x_/TiO_2_ heterojunction can also be fitted by two sets of doublet binding energy peaks associated with W^5+^ and W^6+^ ions, while the peak positions are shift toward the lower-energy side by ~0.4 eV compared to pristine WO_3-x_. Figure 3b shows the high-resolution Ti 2p spectra of pure TiO_2_ and WO_3-x_/TiO_2_. The BE peaks at 458.52 eV and 464.20 eV can be assigned to 2p_3/2_ and 2p_1/2_ of Ti^4+^ in TiO_2_, respectively [46]. In comparison, the Ti 2p peaks of WO_3-x_/TiO_2_ are shifts toward the higher-binding-energy side. The observed changes in the W 4f and Ti 2p binding energies in WO_3-x_/TiO_2_ heterojunction compared to the corresponding values in pristine WO_3-x_ and TiO_2_ imply the formation of intimate interface contact and redistribution of electron density in the vicinity of junction region.

Furthermore, electrical impedance spectroscopy measurements were conducted to analyze the effect of WO_3-x_/TiO_2_ heterojunction on promoting the separation and transfer of charge carriers, which has a significant effect on SERS and photocatalytic properties. As shown in Figure 3c, the Nyquist plot of WO_3-x_/TiO_2_ heterojunction substrates exhibits semicircles with smaller diameters than those of pure WO_3-x_ and TiO_2_, indicating smaller charge transfer resistance, implying reduced recombination of charge carriers in heterojunction samples. Since surface area can also affect SERS and the photocatalytic performance of semiconductor nanostructures, N_2_ adsorption–desorption measurements were carried out on WO_3-x_/TiO_2_ and pure WO_3-x_ (Figure 3d). The extracted specific surface areas are 75.11 and 71.37 m^2^/g for WT-2 and WO_3-x_, respectively. The comparable specific surface area suggests that the difference in SERS and photocatalytic activity between WO_3-x_/TiO_2_ heterojunction and pure WO_3-x_ does not predominantly originate from the difference in surface area.

### 3.2. SERS Activity

**Figure 4:**
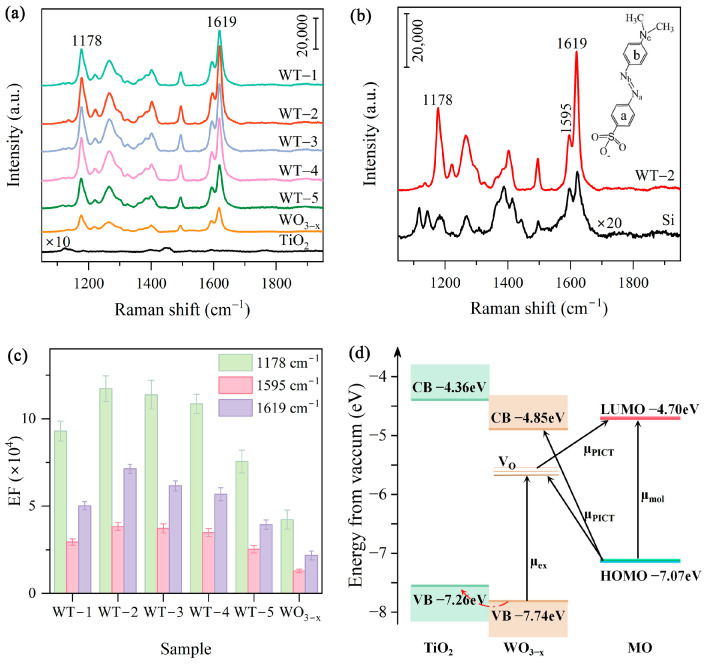
(**a**) SERS spectra of MO (2.5 × 10^−5^ M) on WO_3-x_/TiO_2_ heterojunction substrates prepared at different weight ratios of WCl_6_:TiO_2_. (**b**) Normal Raman spectrum of MO (1.0 × 10^−3^ M) on a silicon wafer (black) together with the SERS spectrum of MO (2.5 × 10^−5^ M) on WT-2 (red). The inset is the molecular structure of MO. (**c**) Calculated enhancement factors. (**d**) Schematic diagram of PICT transition between MO and WO_3-x_/TiO_2_ heterojunction under 532 nm laser excitation.

Figure 4a displayed the SERS spectra of MO (5.0 × 10^−5^ M) adsorbed on WO_3-x_/TiO_2_ heterojunction substrates prepared at different mass ratios of WCl_6_ and P25 and pure WO_3-x_ and TiO_2_. The characteristic vibration peaks of MO molecules can be observed at 1178, 1266, 1402, 1495, 1595 and 1619 cm^−1^. According to previous studies [47,48,49], the two adjacent strong Raman bands 1595 cm^−1^ and 1619 cm^−1^ can be assigned to S-ring and N-ring stretching, respectively; the 1495 cm^−1^ band is related to C-N and C-H bending, the 1402 cm^−1^ band originates from N=N stretching, 1266 cm^−1^ band is associated with C-N and C-C stretching, and 1178 cm^−1^ band is ascribed to C-C stretching and C-C bending (see Table S1). Figure S4 shows the Fourier-transform infrared spectroscopy (FTIR) spectra of bare WO_3-x_/TiO_2_ and the sample collected after 30 min mixing with the MO solution (MO-WO_3-x_/TiO_2_). Comparative analysis reveals two new vibrational bands emerge at 1599 cm^−1^ and 1399 cm^−1^ in MO-WO_3-x_/TiO_2_, corresponding to the C=C stretching vibration and C-H bending vibration modes of methyl orange molecules, respectively [50]. The appearance of these MO-related vibrational modes confirms the successful adsorption of MO molecules onto the surface of the WO_3-x_/TiO_2_ heterojunction substrate.

Compared to pure WO_3-x_, all the heterojunction samples show superior SERS activity, and the SERS intensity of the optimal sample WT-2 is approximately three times that of WO_3-x_. For the case of pure TiO_2_, no pronounced characteristic Raman features associated with MO were detected. These results demonstrate that the construction of WO_3-x_/TiO_2_ type II heterojunction substrates can effectively boost SERS performance. Moreover, the SERS spectra of WO_3-x_/TiO_2_ heterojunction substrates exhibit clear variation in the relative intensity of Raman peaks in comparison with the normal Raman spectrum of MO (1.0 × 10^−3^ M) on silicon substrate (Figure 4b). More specifically, the relative intensity of 1619 cm^−1^ peak (I_1619_) to 1595 cm^−1^ peak (I_1595_) is increased from 1.36 on Si to 2.54 on WO_3-x_/TiO_2_. Moreover, the intensity of 1178 cm^−1^ peak is also substantially increased (I_1178_/I_1595_ from 0.48 to 1.48) and becomes the second strongest peak in the SERS spectra of MO on WO_3-x_/TiO_2_ heterojunction substrates. The selective enhancement of azo dyes has been observed previously and can plausibly be attributed to adsorption orientation based on surface selection rule [5,49]. The SERS spectra of WO_3-x_/TiO_2_ heterojunction and WO_3-x_ shows a similar variation tendency of relative intensities. We may reasonably expect that contribution to SERS enhancement mainly associated with charge transfer transition between and MO and WO_3-x_. The superior SERS activity of WO_3-x_/TiO_2_ to WO_3-x_ can be attributed to reduced recombination of photogenerated charge carriers by construction of a heterojunction with staggered band alignment. In the WO_3-x_/TiO_2_ type II heterojunction, titanium dioxide possesses both higher conduction band (CB) and valence band (VB) energy levels compared to the counterparts of tungsten oxide. As a result, photogenerated electrons in the CB of titanium dioxide can transfer to tungsten oxide, while holes in the VB of tungsten oxide simultaneously transfer to titanium dioxide. The spatial separated charge transfer mechanism effectively suppresses the recombination of electron–hole pairs, which is consistent with the reduced charge transfer resistance observed in the EIS analysis. The suppression of charge recombination in WO_3-x_/TiO_2_ increases the population of available photoinduced charge carriers participating in charge transfer between semiconductor substrate and adsorbed MO molecules, thereby enhancing the SERS performance of the heterojunction samples. The different Raman enhancement abilities of heterojunction samples prepared at various weight ratios observed could be plausibly explained by considering the following factors: (1) these heterojunction samples may have different junction interface area, which will affect interfacial charge separation and transfer in semiconductor nanocomposites; (2) different contents of WO_3-x_ might affect CT between substrate and analyte. Figure 4c shows the enhancement factors (EF), an important parameter of SERS substrate, calculated based on the intensity of 1178 cm^−1^ (I_1178_), 1595 cm^−1^ (I_1595_) and 1619 cm^−1^ (I_1619_) peaks. The following formula is used to calculate EF:

$$x=\frac{I_{SERS}}{N_{SERS}}/\frac{I_{NR}}{N_{NR}}$$

where $I_{SERS}$ is the SERS intensity of MO (5.0 × 10^−5^ M) on WO_3-x_/TiO_2_ heterojunction and WO_3-x_ in Figure 4a and $I_{NR}$ is the normal Raman intensity in Figure 4b, while $N_{SERS}$ and $N_{NR}$ denote, respectively, the number of MO molecules for SERS and normal Raman spectra measurements. The details of calculations are provided in Section S2 of the Supplementary Materials. The optimal sample WT-2 exhibits an evaluated EF of 1.2 × 10^5^. Additionally, a comparative analysis of excitation wavelength on SERS activity was also conducted. Figure S5 presents SERS spectra of MO (5.0 × 10^−5^ M) adsorbed on WT-2 under 532 nm and 785 nm laser excitation. It can be found that WO_3-x_/TiO_2_ heterojunction exhibits a higher enhancement capability under the excitation of 532 nm for MO detection.

To elucidate the mechanism of enhanced SERS performance of WO_3-x_/TiO_2_ heterojunction, a schematic diagram of energy level alignment and facilitated photoinduced charge transfer (PICT) pathways is shown in Figure 4d. The conduction band (CB) edge and the valence band (VB) edge of WO_3-x_ are positioned at −4.85 eV and −7.74 eV with respect to the vacuum level at 0 eV, respectively [51]. In addition, the existence of rich oxygen vacancies (V_O_) gives rise to defect levels located at 0.5−1.0 eV below the bottom of the conduction band, which can serve as a mediator for electrons to migrate between the substrate and the molecule [34]. For Degussa P25, the CB minimum and the VB maximum are positioned at 4.36 eV and 7.26 eV below the vacuum level [52]. As depicted in the energy level diagram in Figure 4d, the conduction band of WO_3-x_ lies 0.49 eV below that of TiO_2_ (i.e., WO_3-x_ has a more negative CB potential relative to the vacuum level), while the valence band of TiO_2_ is 0.48 eV higher than that of WO_3-x_ (i.e., the TiO_2_ VB is less negative compared to WO_3-x_). Thus, photogenerated electrons in the TiO_2_ CB migrate to lower-energy WO_3-x_ CB, while photogenerated holes in the WO_3-x_ VB transfer upward to the higher lying VB of TiO_2_. The opposing transfer direction of electrons and holes leads to a spatial separation of charge carriers, and subsequently reduced recombination. For MO molecules, the lowest unoccupied molecular orbital (LUMO) and the highest occupied molecular orbital (HOMO) are positioned at about 4.70 eV and 7.07 eV below the vacuum level, respectively [53]. It has been previously proposed [34,54] that SERS enhancement of defect-rich semiconductor substrate can be expressed as a sum of two vibronic coupling terms: (1) term B, representing the Raman enhancement contribution of molecule-to-semiconductor CT transitions to the polarizability tensor (including charge transfer from HOMO to CB and CT from HOMO to defect states), and (2) term C, representing the enhancement contribution of semiconductor-to-molecule transitions to the polarizability tensor (including CT from CB to LUMO and CT from defect states to LUMO). The photon energy provided by 532 nm laser is 2.33 eV. Under laser excitation, electrons can be excited from VB of WO_3-x_ to V_O_-related defect levels within the bandgap, followed by a subsequent transfer to the LUMO of MO molecules. This two-step charge transfer process directly contributes to the enhancement in SERS activity through the CM mechanism. However, a competing recombination pathway exists where the V_O_ defect-trapped electrons may recombine with photogenerated holes in the WO_3-x_ VB through Coulombic attraction. This recombination process reduces the number of available electrons which can participate in the charge–transfer transition from WO_3-x_ to MO molecules, thereby reducing the PICT contribution to SERS enhancement. For the WO_3-x_/TiO_2_ heterojunction substrate, the VB of TiO_2_ lies above that of WO_3-x_, creating a thermodynamically favorable pathway for photoexcited VB holes in WO_3-x_ to migrate toward the TiO_2_ VB. The construction of WO_3-x_/TiO_2_ type II heterojunction can effectively suppress the recombination of VB holes and defect-trapped electrons in WO_3-x_. Consequently, a greater proportion of photogenerated electrons remain available to participate in the CT process from WO_3-x_ to MO, enhancing SERS performance. Moreover, electrons can also be excited from the HOMO level of MO to the defect levels in WO_3-x_ with an energy separation of about 1.22 eV and from the HOMO of MO to CB of WO_3-x_ with an energy separation of 2.22 eV. In addition, the electrons on the LUMO of MO could easily migrate to the CB of WO_3-x_ due to the small difference in energy (0.15 eV). The superior SERS activity of WO_3-x_/TiO_2_ to WO_3-x_ demonstrates that inhibition of charge recombination via the fabrication of type II heterojunction is an effective approach to improve PICT, and hence boosting SERS activity.

**Figure 5:**
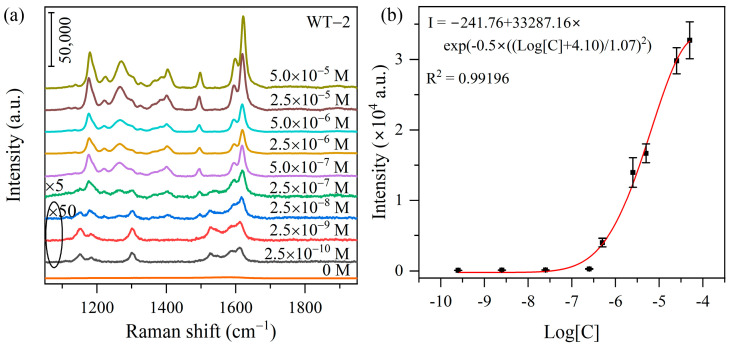
(**a**) SERS spectra on WT-2 heterojunction substrate when the concentration of MO solutions is varied from 5.0 × 10^−5^ M to 2.5 × 10^−10^ M and the Raman spectrum of the bare substrate (0 M). (**b**) GaussAmp nonlinear fitting of 1178 cm^−1^ peak intensity versus logarithm of MO concentration (Log[C]).

Figure 5a shows the SERS spectra of MO at concentrations varying from 10^−5^ M to 10^−10^ M on the WT-2 heterojunction substrate. As a reference, the Raman spectra of bare substrate WT-2 (0 M) was included, which exhibit no detectable Raman vibration peaks in the spectral region, suggesting that the observed SERS features at low concentration of MO originate from the analyte molecule rather than the substrate. Upon dilution, the intensity of SERS signals decreases since fewer MO molecules are adsorbed on the substrates. As shown in Figure 5a and Figure S6, characteristic Raman features of MO molecules remain discernible at 10^−10^ M, demonstrating a reasonably high SERS sensitivity of WO_3-x_/TiO_2_ heterojunction. Figure 5b plots the 1178 cm^−1^ peak intensity versus logarithm of MO concentration (Log[C]), which can be well fitted by a GaussAmp nonlinear relationship with R^2^ = 0.99196. This type of relationship between SERS intensity and logarithm of analyte concentration has been previously reported for metal oxide substrate with rich oxygen vacancies [55]. We would like to point out that the proposed WO_3-x_/TiO_2_ heterojunction exhibits reasonably good sensitivity and enhancing ability compared to some common semiconductor-based SERS substrates subjected to defect engineering, morphology manipulation and heterojunction construction (Table S2).

**Figure 6:**
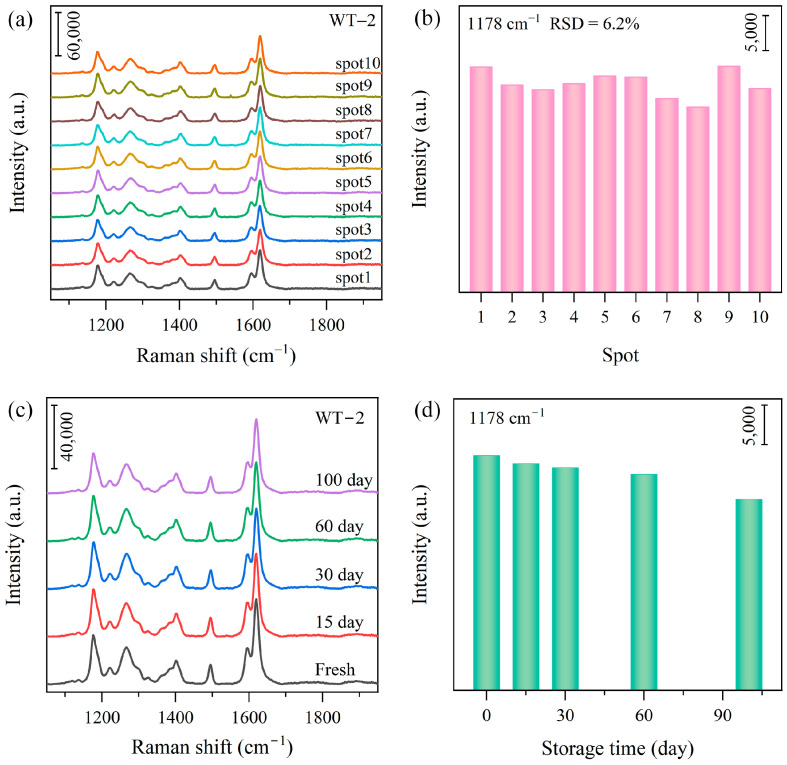
(**a**,**b**) SERS spectra of MO (2.5 × 10^−5^ M) collected from ten different spots on WT-2, and the corresponding 1178 cm^−1^ peak intensity. (**c**,**d**) SERS spectra of WT-2 subjected to storage for a different period of time, and the corresponding 1178 cm^−1^ peak intensity.

In addition, the repeatability and uniformity of the WO_3-x_/TiO_2_ heterojunction SERS substrates are also investigated. Multiple SERS measurements were performed on different regions of WO_3-x_/TiO_2_ heterojunction substrate. As can be seen from Figure 6a,b, SERS spectra collected from different regions of WT-2 substrate show good reproducibility and the calculated relative standard deviation (RSD) is 6.2% for the 1178 cm^−1^ peak. Similarly, other heterojunction SERS substrates also show good uniformity with an RSD below 10% (Figure S7). In addition, the WO_3-x_/TiO_2_ heterojunction substrates were maintained at room temperature, and storage stability was also evaluated. As shown in Figure 6c,d, the SERS spectra of MO on WT-2 barely changed over 100 days and the peak intensity of 1178 cm^−1^ remains 81% that of freshly prepared sample, suggesting long-term stability after storage at room temperature.

### 3.3. Photocatalytic Activity

**Figure 7:**
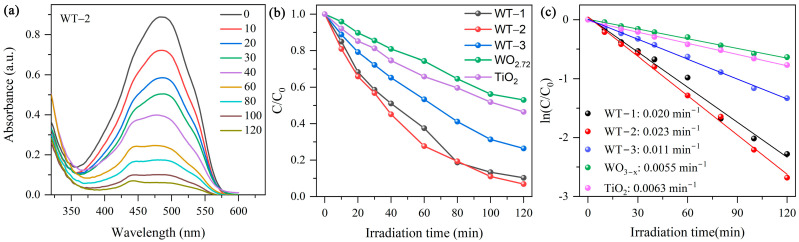
(**a**) UV–visible absorption spectra of MO solutions during MO degradation over WT-2. (**b**) Photodegradation efficiency (C_t_/C_0_) of WO_3-x_/TiO_2_ heterojunction nanocomposites together with pure WO_3-x_ and TiO_2_. (**c**) Fitting of ln(C_t_/C_0_) versus time and the estimated kinetic constant.

In the previous section, we demonstrated the enhancement effect of the WO_3-x_/TiO_2_ type II heterojunction on SERS performance. Since rapid recombination of photo-generated electron–hole pairs is also a known obstacle for limiting photocatalytic performance, it is intriguing to examine the photocatalytic degradation efficiency of WO_3-x_/TiO_2_ heterojunction compared with its component semiconductors. Figure 7a shows the UV-Vis absorption spectra of MO solutions collected during photocatalytic degradation over WT-2. The absorption peak is steadily decreased as the irradiation time is prolonged from 0 min to 120 min, demonstrating gradual decomposition of MO molecules over the WO_3-x_/TiO_2_ heterojunction sample. The estimated photodegradation efficiency (C_t_/C_0_) was plotted in Figure 7b. It can be found that ~93% of MO was decomposed under visible-light illumination for 120 min. Comparatively, pristine WO_3-x_ and TiO_2_ exhibits much lower photodegradation efficiency, and 47% and 54% of MO was decomposed, respectively. Figure 7c plots the −ln(C_t_/C_0_) versus irradiation time, which can be fitted by a linear relationship ln(C/C0) = -kt, where k is the rate constant, indicating the degradation kinetic involves a typical first-order reaction. The pseudo-first-order rate constant (k) value determined stood at 0.020, 0.023, 0.011, 0.0055 and 0.0063 min^−1^ for WT-1, WT-2, WT-3, WO_3-x_ and TiO_2_, respectively. The higher photodegradation efficiency of heterojunction samples is presumably due to reduced recombination of photogenerated charge carriers. Under visible-light irradiation (λ ≥ 400 nm), electron–hole pairs can be generated in both TiO_2_ and WO_3-x_. Based on our previous discussion, photogenerated electrons in CB of TiO_2_ can transfer to WO_3-x_, while photoexcited holes in the VB of WO_3-x_ simultaneously migrate toward TiO_2_ in WO_3-x_/TiO_2_ type II heterojunction photocatalysts with staggered band alignment. Due to the reducing recombination, more photogenerated charge carriers can migrate to catalyst surface and participate in redox reactions. Hence, WO_3-x_/TiO_2_ heterojunction exhibits higher photodegradation efficiency than that of pure WO_3-x_ and TiO_2_. Since the standard redox potential of ·OH/OH^−^ is −6.49 eV (vs. vacuum level) [56], it is thermodynamically favorable for holes in a semiconductor, with VB lying below this redox potential to generate ·OH. With VB positioned at −7.26 eV, photogenerated holes accumulated in TiO_2_ can migrate to the surface and contribute to photodegradation by either producing ·OH or by direct oxidation of MO. Regarding to heterojunction samples with a different content of WO_3-x_ and TiO_2_, such dependence of photocatalytic performance on component proportions is frequently observed for heterojunction photocatalysts. A plausible explanation is that nanocomposite photocatalysts contain multiple interfaces between individual components, and the variation in their proportions could change the total area of intimate contact, affecting the transfer and separation of photogenerated charge carriers. This is consistent with the experimental results that the heterojunction photocatalyst’s higher degradation efficiency exhibits a smaller diameter of semicircle in the Nyquist plot (Figure 3c), suggesting that the improved separation and transfer of photoinduced charge carriers play a key role on the improved photocatalytic activity of WO_3-x_/TiO_2_.

**Figure 8:**
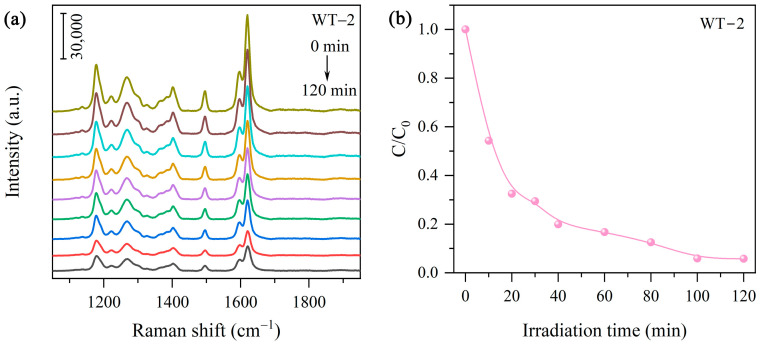
(**a**) SERS spectra of MO solutions withdrawn at given time intervals during MO degradation over WT-2. (**b**) Photodegradation efficiency estimated based on SERS measurement.

In addition to UV-Vis absorption spectroscopy, SERS was utilized to monitor the photocatalytic process. Since the SERS intensity of MO on WO_3-x_/TiO_2_ heterojunction remains relatively high even as the MO concentration is down to 10^−7^ M (Figure 5a), SERS emerges as an appealing analytical technique to monitor photodegradation activity. Figure 8a showed the SERS spectra measured during the photodegradation process of MO over WT-2. With increasing irradiation time, the SERS intensity of the characteristic vibration modes of MO is gradually decreased. At a given time t, the MO concentration C_t_ is extracted from the extracted empirical relationship between SERS intensity and MO presented in Figure 5b, and the evaluated photodegradation efficiency C/C_0_ is shown in Figure 8b. It can be found that ~94% decomposition of MO occurred within 120 min, which is in good agreement with the result obtained from the traditional UV-Vis absorption spectroscopy technique.

**Figure 9:**
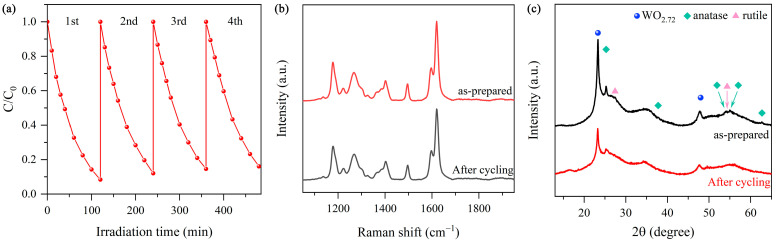
(**a**) Results of consecutive photocatalytic experiments. (**b**) SERS spectrum of WT-2 before and after photocatalytic cycling experiments. (**c**) XRD before and after photocatalytic experiments.

As shown in Figure 9a, the photocatalytic activity of WT-2 remains 90% that of the freshly prepared sample after four consecutive runs. Notably, the SERS signal intensity of WT-2 after the cycling experiments remains 87% that of the freshly prepared sample (Figure 9b). Compared to the freshly prepared sample, there is no pronounced change in the XRD pattern of WT-2 collected after four consecutive runs (Figure 9b). These results demonstrate that WO_3-x_/TiO_2_ heterojunction samples have excellent stability and are suitable for re-utilization.

## 4. Conclusions

In summary, we have demonstrated an efficient SERS substrate through the construction of a WO_3-x_/TiO_2_ heterojunction with staggered band alignment. The optimized sample exhibits the LOD of 10^−10^ M and EF of 1.2 × 10^5^ for methyl orange. The excellent SERS performance can be attributed to improved separation and transfer of photoinduced electron–hole pairs, and consequently, more charge carriers can contribute to the charge–transfer process between SERS substrates and analyte molecules. Moreover, rich oxygen vacancies in WO_3-x_ result in defect levels in forbidden gap and provide extra CT pathways, which are beneficial for improving SERS activity. Thereafter, WO_3-x_/TiO_2_ heterojunction samples were used to degrade MO under visible-light illumination and exhibit superior photodegradation efficiency compared to that of pure WO_3-x_ and TiO_2_. Given the relatively high signal intensity even when the MO concentration is reduced below the micromolar level, SERS is employed to monitor the photocatalytic process and the estimated photodegradation efficiency is comparable to that obtained from transitional UV-Vis spectroscopy. Furthermore, the WO_3-x_/TiO_2_ heterojunction exhibits excellent stability for dual-function applications of SERS sensing and photocatalysis. Specifically, the SERS activity of WO_3-x_/TiO_2_ heterojunction remains 81% that of the freshly prepared sample after storage at room temperature for up to 100 days. Over four consecutive runs of photodegradation experiments, the photocatalytic efficiency of WO_3-x_/TiO_2_ heterojunction can remain 90% that of the freshly prepared sample. Meanwhile, the SERS intensity of the recycled WO_3-x_/TiO_2_ heterojunction is maintained at 87%. Hence, this work provides insightful information on the development of low-cost and stable dual-function materials for SERS sensing and photodegradation of organic pollutants.

## Data Availability

All data are contained within this article.

## Supplementary Materials

The following supporting information can be downloaded at: https://www.mdpi.com/article/10.3390/nano15070521/s1, Figure S1: (a) XRD curve of pure WO_3-x_ and standard pattern of WO_2.72_ in monoclinic phase (PDF card No. 73-2177). (b) XRD curve of Degussa P25 and standard patterns of TiO_2_ in anatase phase (PDF card No. 21-1272) and rutile phase (PDF No. 21-1276). (c,d) Raman spectra of pure WO_3-x_ and Degussa P25, respectively; Figure S2: XPS survey spectra of (a) WO_3-x_, (b) TiO_2_ and (c) WO_3-x_/TiO_2_; Figure S3: High resolution O 1s spectra of (a) WO_3-x_, and (b) WO_3-x_/TiO_2_; Figure S4: FTIR spectrum of WO_3-x_/TiO_2_ before and after adsorption of MO; Figure S5: SERS spectra of MO (2.5 × 10^−5^ M) on WT−2 under 532 nm laser excitation green, laser power = 0.1 mW, exposure = 3, accumulation = 3) and 785 nm laser excitation (green, laser power = 0.6 mW, exposure = 12, accumulation = 9); Figure S6: Magnified comparison of SERS spectra at 2.5 × 10^−10^ M MO (green), 2.5 × 10^−6^ M (orange) and the bare WT-2 heterojunction substrate (blue); Figure S7: (a,c,e,g) SERS spectra of MO (2.5 × 10^−5^ M) on WT-1, WT-3, WT-4, and WT-5 collected from ten different spots, respectively. (b,d,f,h) Corresponding peak intensity at 1178 cm^−1^; Table S1: Characteristic Raman peak positions of MO adsorbed on WO_3-x_/TiO_2_ heterojunction and silicon wafer, and their tentative assignment; Table S2: Summary of different semiconductor-based SERS substrates. See Refs [23,28,31,34,41,47,48,49,57,58,59,60,61,62,63,64,65,66]

## References

[1] Yi J., You E.-M., Hu R., Wu D.-Y., Liu G.-K., Yang Z.-L., Zhang H., Gu Y., Wang Y.-H., Wang X. (2025). Surface-enhanced Raman spectroscopy: A half-century historical perspective. Chem. Soc. Rev..

[2] Jiao A., Cui Q., Li S., Li H., Xu L., Tian Y., Ma H., Zhang M., Liu X., Chen M. (2022). Aligned TiO_2_ nanorod arrays decorated with closely interconnected Au/Ag nanoparticles: Near-infrared SERS active sensor for monitoring of antibiotic molecules in water. Sens. Actuators B Chem..

[3] Nilghaz A., Mahdi Mousavi S., Amiri A., Tian J., Cao R., Wang X. (2022). Surface-Enhanced Raman Spectroscopy Substrates for Food Safety and Quality Analysis. J. Agric. Food Chem..

[4] Zappalà G., Soufi G., Dumont E., Molander N., Slipets R., Thamdrup L.H.E., Andersson P.O., Rindzevicius T., Boisen A. (2025). SERS-integrated centrifugal microfluidic platform for the detection and quantification of Chemical Warfare Agents in single-component solution and mixtures. Sens. Actuators B Chem..

[5] Itoh T., Procházka M., Dong Z.-C., Ji W., Yamamoto Y.S., Zhang Y., Ozaki Y. (2023). Toward a New Era of SERS and TERS at the Nanometer Scale: From Fundamentals to Innovative Applications. Chem. Rev..

[6] Ding S.-Y., You E.-M., Tian Z.-Q., Moskovits M. (2017). Electromagnetic theories of surface-enhanced Raman spectroscopy. Chem. Soc. Rev..

[7] Langer J., Jimenez de Aberasturi D., Aizpurua J., Alvarez-Puebla R.A., Auguié B., Baumberg J.J., Bazan G.C., Bell S.E.J., Boisen A., Brolo A.G. (2020). Present and Future of Surface-Enhanced Raman Scattering. ACS Nano.

[8] Kim J.-M., Kim J., Choi K., Nam J.-M. (2023). Plasmonic Dual-Gap Nanodumbbells for Label-Free On-Particle Raman DNA Assays. Adv. Mater..

[9] Sun L., Cao C., Zhi Y., Shan Y., Zhang H., Dou B., Zhang L., Huang W. (2023). Au–Ag Nanoparticles with Controllable Morphologies for the Surface-Enhanced Raman Scattering Detection of Trace Thiram. ACS Appl. Nano Mater..

[10] Anantha P., Raj P., Zheng P., Tanwar S., Barman I. (2025). Gold nanoprism enhanced SERS aptasensor for simultaneous detection of thrombin and VEGF. Sens. Actuators B Chem..

[11] Husain S., Mutalik C., Yougbaré S., Chen C.-Y., Kuo T.-R. (2024). Plasmonic Au@Ag Core–Shell nanoisland Film for Photothermal Inactivation and Surface-Enhanced Raman Scattering Detection of Bacteria. Nanomaterials.

[12] Kaja S., Nag A. (2021). Bimetallic Ag–Cu Alloy Microflowers as SERS Substrates with Single-Molecule Detection Limit. Langmuir.

[13] Lin J., Hao W., Shang Y., Wang X., Qiu D., Ma G., Chen C., Li S., Guo L. (2018). Direct Experimental Observation of Facet-Dependent SERS of Cu_2_O Polyhedra. Small.

[14] Qi D., Lu L., Wang L., Zhang J. (2014). Improved SERS Sensitivity on Plasmon-Free TiO_2_ Photonic Microarray by Enhancing Light-Matter Coupling. J. Am. Chem. Soc..

[15] Singh N.S., Mayanglambam F., Nemade H.B., Giri P.K. (2022). Plasma-Treated Graphene Surfaces for Trace Dye Detection Using Surface-Enhanced Raman Spectroscopy. ACS Appl. Nano Mater..

[16] Wu Y., Sun T., Shao M., Ji C., Li C., Zhang C., Li Z. (2024). Pyroelectrically Driven Charge Transfer and its Advantages on SERS and Self-Cleaning Property. Laser Photonics Rev..

[17] Jiang Y., Cong S., Song G., Sun H., Zhang W., Yao W., Zhao Z. (2021). Defective cuprous oxide as a selective surface-enhanced Raman scattering sensor of dye adulteration in Chinese herbal medicines. J. Raman Spectrosc..

[18] He R., Lai H., Wang S., Chen T., Xie F., Chen Q., Liu P., Chen J., Xie W. (2020). Few-layered vdW MoO_3_ for sensitive, uniform and stable SERS applications. Appl. Surf. Sci..

[19] Qiu Y., Lin M., Chen G., Fan C., Li M., Gu X., Cong S., Zhao Z., Fu L., Fang X. (2019). Photodegradable CuS SERS Probes for Intraoperative Residual Tumor Detection, Ablation, and Self-Clearance. ACS Appl. Mater. Interfaces.

[20] Pan T., Song G., Cong S., Chen Z., Chen J., Zhao Z. (2020). Hydroxyl Group-Abundant TiO_2_ Semiconductor SERS Sensor toward Polymerization Inhibitor Sensing. J. Phys. Chem. C.

[21] Alessandri I., Lombardi J.R. (2016). Enhanced Raman Scattering with Dielectrics. Chem. Rev..

[22] Ushkov A., Dyubo D., Belozerova N., Kazantsev I., Yakubovsky D., Syuy A., Tikhonowski G.V., Tselikov D., Martynov I., Ermolaev G. (2025). Tungsten Diselenide Nanoparticles Produced via Femtosecond Ablation for SERS and Theranostics Applications. Nanomaterials.

[23] Liu W., He X., Wang Z., Yuan M., Zhao Z., Ye X., Shang S., Song Z., Huang L., Liu Y. (2024). Geometric and Electronic Structure Modulation to Optimize the Charge Transfer of TiO_2_ for Ultrasensitive and Stable SERS Sensing. Inorg. Chem..

[24] Xu J., Shi X., Yi M., Chi Y., Mao Z., Yang B., Jung Y.M. (2025). Lithium-doped ZrO_2_ nanoparticles for SERS-based norfloxacin drug detection. Spectrochim. Acta Part A.

[25] Cao Y., Liang P., Dong Q., Wang D., Zhang D., Tang L., Wang L., Jin S., Ni D., Yu Z. (2019). Facile Reduction Method Synthesis of Defective MoO_2–x_ Nanospheres Used for SERS Detection with High Chemical Enhancement. Anal. Chem..

[26] Sun H., Wang X., Wu P., Jiang H., Tang J. (2022). Nonstoichiometric tungsten oxide nanosheets with abundant oxygen vacancies for defects-driven SERS sensing. J. Raman Spectrosc..

[27] Quan Y., Tang X.-H., Lu W., Huo W., Song Z., Shen W., Yang M., Huang X.-J., Liu W.-Q. (2023). Amorphous/Crystal Heterostructure Coupled Oxygen Vacancies-Sensitized TiO_2_ with Conspicuous Charge-Transfer Resonance for Efficient SERS Detection of Chloramphenicol. Adv. Opt. Mater..

[28] Jiang X., Xu L., Ji W., Wang W., Du J., Yang L., Song W., Han X., Zhao B. (2022). One plus one greater than Two: Ultrasensitive Surface-Enhanced Raman scattering by TiO_2_/ZnO heterojunctions based on Electron-Hole separation. Appl. Surf. Sci..

[29] He C., Jiang L., Yuan R., Yang X. (2023). Facet junction of CeO_2_ with high SERS activity for sensitive detection of ATP. Sens. Actuators B Chem..

[30] Meng W., Kragt A.J.J., Gao Y., Brembilla E., Hu X., van der Burgt J.S., Schenning A.P.H.J., Klein T., Zhou G., van den Ham E.R. (2024). Scalable Photochromic Film for Solar Heat and Daylight Management. Adv. Mater..

[31] Wang P., Guo S., Hu Z., Li T., Pu S., Mao H., Cai H., Zhu Z., Li H.-Y., Liu H. (2023). W_18_O_49_ sensitized with Pd nanoparticles for ultrasensitive ppb-level formaldehyde detection. Chem. Eng. J..

[32] Zhang Z., Jiang X., Liu B., Guo L., Lu N., Wang L., Huang J., Liu K., Dong B. (2018). IR-Driven Ultrafast Transfer of Plasmonic Hot Electrons in Nonmetallic Branched Heterostructures for Enhanced H_2_ Generation. Adv. Mater..

[33] Wang S.-B., Zhang C., Ye J.-J., Zou M.-Z., Liu C.-J., Zhang X.-Z. (2020). Near-Infrared Light Responsive Nanoreactor for Simultaneous Tumor Photothermal Therapy and Carbon Monoxide-Mediated Anti-Inflammation. ACS Cent. Sci..

[34] Cong S., Yuan Y., Chen Z., Hou J., Yang M., Su Y., Zhang Y., Li L., Li Q., Geng F. (2015). Noble metal-comparable SERS enhancement from semiconducting metal oxides by making oxygen vacancies. Nat. Commun..

[35] Ali S., Ismail P.M., Khan M., Dang A., Ali S., Zada A., Raziq F., Khan I., Khan M.S., Ateeq M. (2024). Charge transfer in TiO_2_-based photocatalysis: Fundamental mechanisms to material strategies. Nanoscale.

[36] Zhu Y., Hao Q., Zhu H., Zhao R., Feng L., He S., Wang W., He G., Liu B., Yang P. (2024). Thermoelectric Nanoheterojunction-Mediated Multiple Energy Conversion for Enhanced Cancer Therapy. ACS Nano.

[37] Jiang M., Yang Z., Lu T., Liu X., Li J., Wang C., Yang G., Pan L. (2024). Machine learning accelerated study for predicting the lattice constant and substitution energy of metal doped titanium dioxide. Ceram. Int..

[38] Zheng Q., Wang T., Li B., Gao R., Zhang X., Cheng X., Huo L., Major Z., Xu Y. (2023). Crosslinked WO_3_ nanonet for rapid detection of sulfur mustard gas simulant: Mechanism insights and sensing application. Sens. Actuators B Chem..

[39] Shiraishi Y., Hirakawa H., Togawa Y., Sugano Y., Ichikawa S., Hirai T. (2013). Rutile Crystallites Isolated from Degussa (Evonik) P25 TiO_2_: Highly Efficient Photocatalyst for Chemoselective Hydrogenation of Nitroaromatics. ACS Catalysis.

[40] Daniel M.F., Desbat B., Lassegues J.C., Gerand B., Figlarz M. (1987). Infrared and Raman study of WO_3_ tungsten trioxides and WO_3_ xH_2_O tungsten trioxide tydrates. J. Solid State Chem..

[41] Zhang W., Yuan T., Wang X., Cheng Z., Xu J. (2022). Coal mine gases sensors with dual selectivity at variable temperatures based on a W_18_O_49_ ultra-fine nanowires/Pd@Au bimetallic nanoparticles composite. Sens. Actuators B Chem..

[42] Yan J., Wang T., Wu G., Dai W., Guan N., Li L., Gong J. (2015). Tungsten Oxide Single Crystal Nanosheets for Enhanced Multichannel Solar Light Harvesting. Adv. Mater..

[43] Mikami M., Nakamura S., Kitao O., Arakawa H. (2002). Lattice dynamics and dielectric properties of TiO_2_ anatase: A first-principles study. Phys. Rev. B.

[44] García-Contreras L.A., Flores-Flores J.O., Arenas-Alatorre J.Á., Chávez-Carvayar J.Á. (2022). Synthesis, characterization and study of the structural change of nanobelts of TiO_2_ (H_2_Ti_3_O_7_) to nanobelts with anatase, brookite and rutile phases. J. Alloy. Compd..

[45] Ma T., Li B., Tian S., Qian J., Zhou L., Liu Q., Liu B., Zhao X., Sankar G. (2023). Reversible photochromic W_18_O_49_: Mechanism revealing and performance improvement for smart windows. Chem. Eng. J..

[46] Zhao J., Liu L., Zhang Y., Feng Z., Zhao F., Wang W. (2021). Light-responsive color switching of self-doped TiO_2−x_/WO_3_·0.33H_2_O hetero-nanoparticles for highly efficient rewritable paper. Nano Res..

[47] Prakash O., Kumar S., Singh P., Deckert V., Chatterjee S., Ghosh A.K., Singh R.K. (2016). Surface-enhanced Raman scattering characteristics of CuO: Mn/Ag heterojunction probed by methyl orange: Effect of Mn^2+^ doping. J. Raman Spectrosc..

[48] Zhang A., Fang Y. (2007). Adsorption orientations and interactions of methyl orange on negatively and positively charged colloidal silver particles. J. Colloid Interface Sci..

[49] Yu T.-H., Ho C.-H., Wu C.-Y., Chien C.-H., Lin C.-H., Lee S. (2013). Metal–organic frameworks: A novel SERS substrate. J. Raman Spectrosc..

[50] Galindo C., Jacques P., Kalt A. (2000). Photodegradation of the aminoazobenzene acid orange 52 by three advanced oxidation processes: UV/H_2_O_2_, UV/TiO_2_ and VIS/TiO_2_: Comparative mechanistic and kinetic investigations. J. Photochem. Photobiol. A.

[51] Deng Y., Tang L., Feng C., Zeng G., Chen Z., Wang J., Feng H., Peng B., Liu Y., Zhou Y. (2018). Insight into the dual-channel charge-charrier transfer path for nonmetal plasmonic tungsten oxide based composites with boosted photocatalytic activity under full-spectrum light. Appl. Catal. B Environ. Energy.

[52] Gong Y., Wu Y., Xu Y., Li L., Li C., Liu X., Niu L. (2018). All-solid-state Z-scheme CdTe/TiO_2_ heterostructure photocatalysts with enhanced visible-light photocatalytic degradation of antibiotic waste water. Chem. Eng. J..

[53] Liu C.-S., Li B.-H., Chen C.-H., Peng J.-W., Lee S. (2014). Enhancement in SERS intensities of azo dyes adsorbed on ZnO by plasma treatment. J. Raman Spectrosc..

[54] Lombardi J.R., Birke R.L. (2014). Theory of Surface-Enhanced Raman Scattering in Semiconductors. J. Phys. Chem. C.

[55] Lin J., Shang Y., Li X., Yu J., Wang X., Guo L. (2017). Ultrasensitive SERS Detection by Defect Engineering on Single Cu_2_O Superstructure Particle. Adv. Mater..

[56] Li Y.-N., Chen Z.-Y., Wang M.-Q., Zhang L.-Z., Bao S.-J. (2018). Interface engineered construction of porous g-C_3_N_4_/TiO_2_ heterostructure for enhanced photocatalysis of organic pollutants. Appl. Surf. Sci..

[57] Wang X., Shi W., Jin Z., Huang W., Lin J., Ma G., Li S., Guo L. (2017). Remarkable SERS Activity Observed from Amorphous ZnO Nanocages. Angew. Chem. Int. Edit..

[58] Zarei A., Shafiekhani A. (2020). Surface-enhanced Raman scattering (SERS) of Methyl Orange on Ag-DLC nanoparticles. Mater. Chem. Phys..

[59] Lin J., Yu J., Akakuru O.U., Wang X., Yuan B., Chen T., Guo L., Wu A. (2020). Low temperature-boosted high efficiency photo-induced charge transfer for remarkable SERS activity of ZnO nanosheets. Chem. Sci..

[60] Zheng Z., Cong S., Gong W., Xuan J., Li G., Lu W., Geng F., Zhao Z. (2017). Semiconductor SERS enhancement enabled by oxygen incorporation. Nat. Commun..

[61] Zhang J., Xing T., Zhang M., Zhou Y. (2022). Facile preparation of Cu_2-x_S supernanoparticles with an unambiguous SERS enhancement mechanism. Chem. Eng. J..

[62] Zhou L., Zhou J., Lai W., Yang X., Meng J., Su L., Gu C., Jiang T., Pun E.Y.B., Shao L. (2020). Irreversible accumulated SERS behavior of the molecule-linked silver and silver-doped titanium dioxide hybrid system. Nat. Commun..

[63] Wei J., Qayum A., Jiao X., Wang T., Chen D. (2021). Photo-reduced WO_3_/PAN nanofiber membranes with deposited Ag nanoparticles as efficient SERS substrates. Appl. Surf. Sci..

[64] Yin W., An S., Cheng T., Jiang L., Cao Y. (2024). Enhancing SERS sensitivity of semiconductors through constructing CuO@TiO_2_ heterojunctions via atomic layer deposition. Appl. Surf. Sci..

[65] Wei J., Yu K., Yu Y., Li S., Yu H., Li B., Cui Y., Abdul Q., Chen Q., Hao Z. (2024). Photo-reduced TiO_2_@WO_3_ electrospun nanofibers for efficient SERS and photoelectrochemical performances. Compos. Commun..

[66] Korkmaz I., Sakir M., Sarp G., Salem S., Torun I., Volodkin D., Yavuz E., Onses M.S., Yilmaz E. (2021). Fabrication of superhydrophobic Ag@ZnO@Bi_2_WO_6_ membrane disc as flexible and photocatalytic active reusable SERS substrate. J. Mol. Struct..

