# Supplementary material for: Development of Defect-Rich WO3-x/TiO2 Heterojunction Toward Dual-Functional Enhancement: Boosting SERS and Photocatalytic Performance
Source: Nanomaterials (Basel). 2025 Mar 30;15(7):521. doi: 10.3390/nano15070521 (PMC11990290; doi:10.3390/nano15070521)
Supplement: Supplementary file 1 [file nanomaterials-15-00521-s001.zip › nanomaterials-3508071-supplementary.pdf]

Supplementary Information for

Development of Defect-Rich WO_{3-x}/TiO_2 Heterojunction toward Dual-Functional Enhancement: Boosting SERS and Photocatalytic Performance

Xunfei He, Yinyan Gong*, Lengyuan Niu, and Can Li

Institute of Optoelectronic Materials and Devices, College of Optical and Electronic Technology, China Jiliang University, Hangzhou, Zhejiang 310020, China

Email address: 13A0502075@cjlu.edu.cn

S1. Material Characterization

The structural and morphological properties of WO_{3-x}/TiO_2 heterojunction nanocomposites were characterized by X-ray diffraction (XRD; Rigaku SmartLab, Japan), Raman spectroscopy (Renishaw inVia, UK), and transmission electron microscopy (TEM; FEI Tecnai G2 F20, Japan) measurements. Moreover, the chemical composition and valence states were analyzed by X-ray photoelectron spectroscopy (XPS; Thermo Fisher ESCALAB 250Xi, Al Kα) and the spectra were calibrated with respected to C 1s component at 284.8 eV. N_2 adsorption–desorption measurements were conducted on a Micromeritics Tristar II 3020 physisorption analyzer and the specific surface area was extracted based on Brunauer–Emmett–Teller (BET) equation. Electrochemical impedance spectroscopy (EIS) measurements were carried out on a electrochemical workstation (Chenhua CHI660E) in 1.0 M KOH solution in a three-electrode configuration. The working electrode was prepared by mixing sample powder, Nafion, ethanol, and deionized water (5 mg: 20 μL: 240 μL: 240 μL) thoroughly and drop-casting on a clean nickel foam to form a working area of 1 cm × 1 cm. A platinum foil and a mercuric oxide electrode were used as counter and reference electrodes, respectively. Ultraviolet–Visible (UV–Vis) absorption spectra were recorded on a

Lamada 750S (Perkin Elmer, US) and a 722s UV–Vis spectrometer (Jingke, Shanghai, China).

S2. Enhancement Factor

The enhancement factor (EF) is calculated using the following equation [1]:

$$\mathrm{EF}=\frac{I_{\mathrm{SERS}}/N_{\mathrm{SERS}}}{I_{\mathrm{Raman}}/N_{\mathrm{Raman}}} = \frac{I_{\mathrm{SERS}}}{I_{Raman}} \times \frac{N_{\mathrm{Raman}}}{N_{SERS}} \qquad (1)$$

where I_{SERS} and I_{Raman} refer to the Raman scattering intensities of the characteristic peaks from MO molecules on WO_{3-x}/TiO_2 and silicon wafer, respectively. N_{SERS} and N_{Raman} represent the number of MO molecules contributing to SERS and normal Raman signals, respectively. For normal Raman measurement (Figure 4b), methyl orange (MO) solution (50 μL, 1.0×10^{-3} M) was dropped on the surface of silicon wafer, and dried. A spot with an area (S) approximately 0.17 cm^2 was formed. N_{Raman} can be estimated based on the following equation:

$$\mathrm{N_{Raman}} = \frac{\mathrm{C\times V\times S_{beam}}}{\mathrm{S}} \times \mathrm{N_{AV}} \qquad (2)$$

where C is the concentration of MO (C = 1.0×10^{-3} M), V is the volume of MO solution dropped on the surface of silicon chip (V= 50 μL), and N_{AV} is the Avogadro's constant. S_{beam} is the probing area under laser illumination and is estimated by:

$$\mathrm{S_{beam}}=\pi\times\left(\frac{1.22\times\lambda}{2NA}\right)^2 \qquad (3)$$

where λ = 532 nm and numerical aperture of the objective lens NA = 0.75.

For SERS measurements, N_{SERS} can be calculated using equation:

$$\mathrm{N_{SERS}} = \frac{\mathrm{S_{beam}}}{\mathrm{S_{MO}}} \qquad (4)$$

S_{MO} is the cross-sectional area per MO molecule (~0.935 nm^2) [2]. Using WT−2 as an example, I_{SERS} and I_{Raman} of the characterstic MO vibration mode at 1178 cm^{-1} equal to 30110 and 421, respectively (Figure 4a). Accordingly, the value of EF is calculated to be 1.2×10^5. The same procedures were carried out to calculate EFs of other samples and other Raman modes of MO, and the obtained results are presented in Figure 4c.

S3. Supplementary Figures

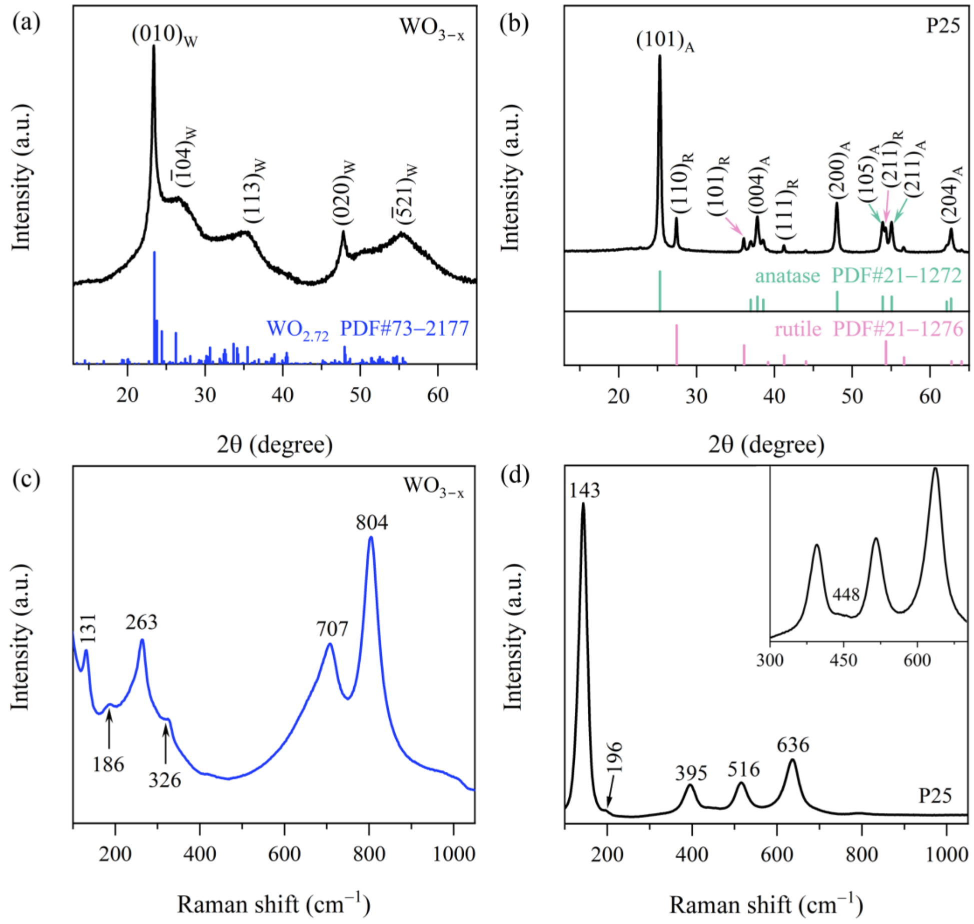

Figure S1. (a) XRD curve of pure WO_{3-x} and standard pattern of $WO_{2.72}$ in monoclinic phase (PDF card no. 73–2177). (b) XRD curve of Degussa P25 and standard patterns of TiO_2 in anatase phase (PDF card no. 21–1272) and rutile phase (PDF no. 21–1276). Diffraction peaks originated from anatase and rutile were labels by subscript A and R, respectively. (c, d) Raman spectra of pure WO_{3-x} and Degussa P25, respectively.

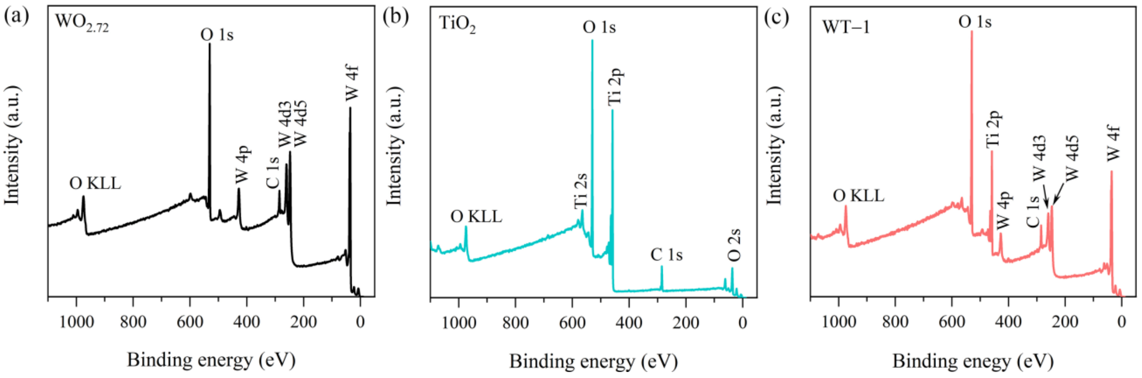

Figure S2. XPS survey spectra of (a) WO_{3-x}, (b) TiO_2 and (c) WO_{3-x}/TiO_2.

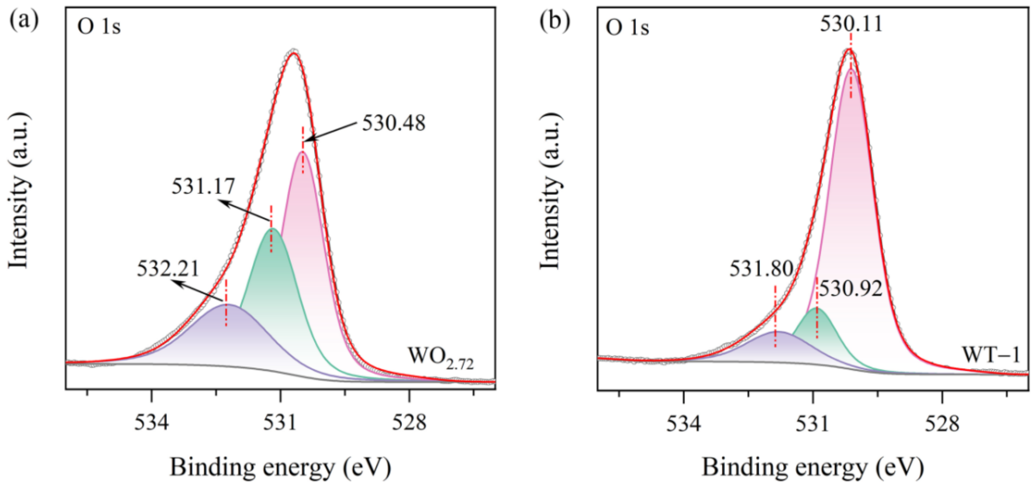

Figure S3. High resolution O 1s spectra of (a) WO_{3-x}, and (b) WO_{3-x}/TiO_2.

The O 1s spectra in Fig. S3 can be fitted into three peaks centered around 530.48 eV, 531.17 eV and 532.21 eV, which are ascribed to the lattice oxygen species, the oxygen species adsorbed on surface vacancies and the surface adsorbed oxygen species, respectively [3,4].

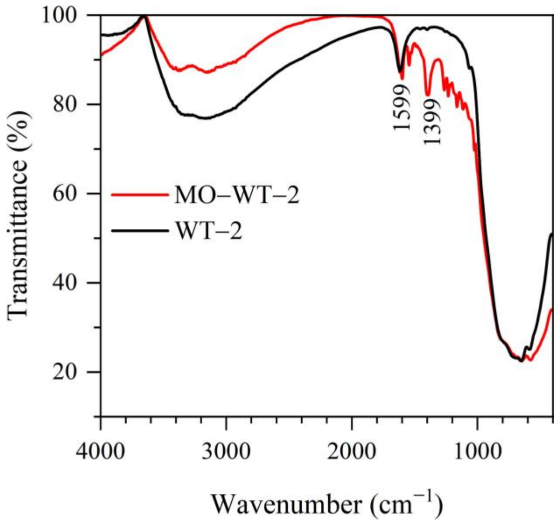

Figure S4. FTIR spectrum of WO_{3-x}/TiO_2 before and after adsorption of MO.

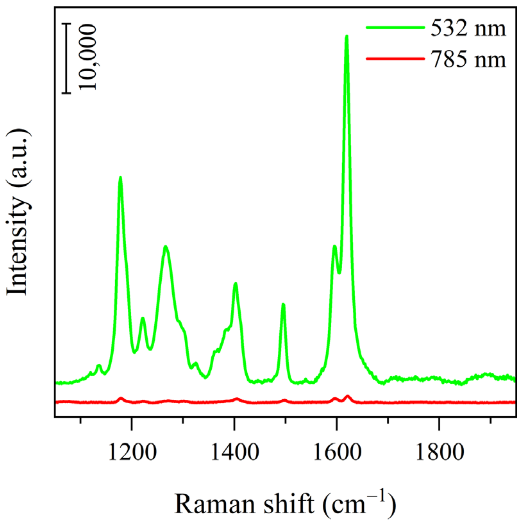

Figure S5. SERS spectra of MO (2.5×10^{-5} M) on WT−2 under 532 nm laser excitation green, laser power = 0.1 mW, exposure = 3, accumulation = 3) and 785 nm laser excitation (green, laser power = 0.6 mW, exposure = 12, accumulation = 9).

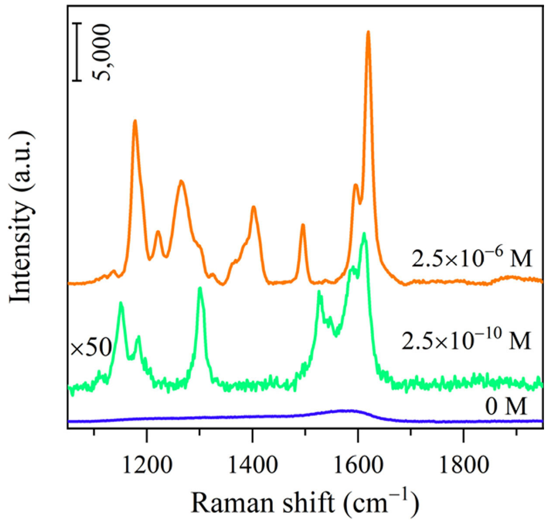

Figure S6. Magnified comparison of SERS spectra at 2.5×10^{-10} M MO (green), 2.5×10^{-6} M (orange) and the bare WT–2 heterojunction substrate (blue).

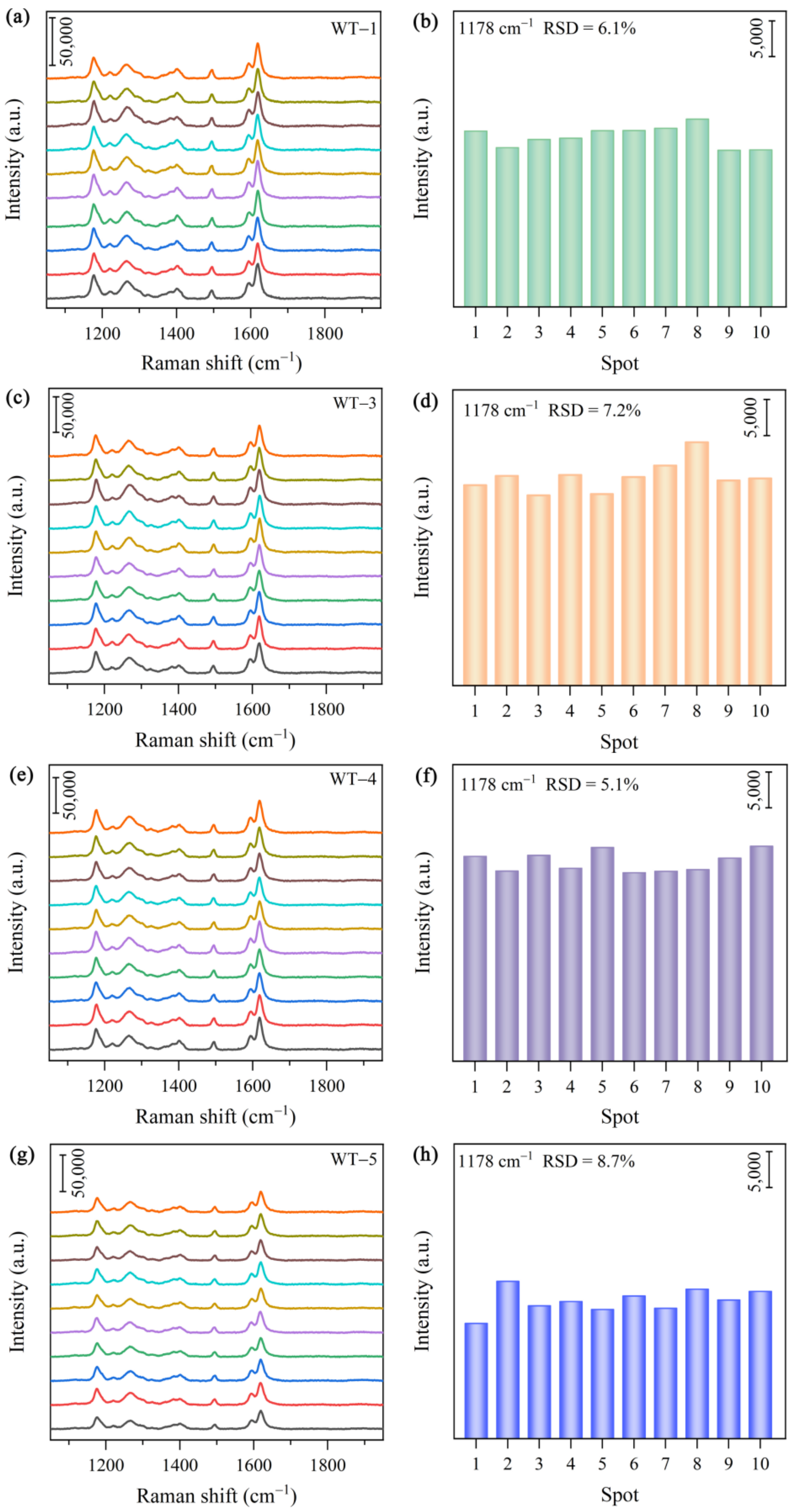

Figure S7 (a, c, e, g) SERS spectra of MO (2.5×10^{-5} M) on WT–1, WT–3, WT–4, and WT–5 collected from ten different spots, respectively. (b, d, f, h) Corresponding peak intensity at 1178 cm^{-1}.

Table S1. Characteristic Raman peak positions of MO adsorbed on WO_{3-x}/TiO_2 heterojunction and silicon wafer, and their tentative assignment.[5-7]

WO_{3-x}/TiO_2 (cm^{-1})	Normal Raman (cm^{-1})	Assignment[a]
	1117	ν(C–)SO_2(–O)
	1144	ν(Ph–N), ν(C–C)
1178	1182	ν(C–C), δ(C–C)
1266	1268	ν(C–N)$_{Me}$, ν(C–C)
1364 (shoulder)	1367 (shoulder)	ν(Ph–N), ν(C–C)
1385 (shoulder)	1389	ν(C–)SO_2(–O), ν(N=N)
1403	1415	ν(N=N)
	1442	ν(C–C), δ(C–H)
1495	1497	τ(C–N)$_{Me}$, δ(C–H)$_{Me}$
1595	1597	ν(C=C) of S ring
1619	1622	ν(C=C) of N ring, N–H in–plane deformation

[a]ν, stretching; δ, bending; τ, torsion; S ring, benzene ring bearing the sulfonate group; N ring, benzene ring bearing aminodimethyl group.

Table S2. Summary of different semiconductor-based SERS substrates.

Material	Analyte	EF	LOD (M)	Excitation (nm)	Reference
Yb-doped TiO_2	4−MBA	/	1×10^{-9}	532	[8]
H_2-treated $W_{18}O_{49}$ nanowire	R6G	3.4×10^{5}	10^{-7}	532	[9]
Low temperature-boosted porous ZnO nanosheet	4−MPY	7.7×10^{5}	1×10^{-7}	532	[10]
Oxygen-incorporated MoS_2 nanosheet	R6G	1.3×10^{5}	1×10^{-7}	532	[11]
$Cu_{2-x}S$ supernanoparticles	R6G	5.34×10^{5}	10^{-8}	532	[12]
Ag/Ag−doped TiO_2	4−MBA	2.9×10^{5}	/	785	[13]
Ag/WO_3/PAN	R6G	2.3×10^{5}	/	532	[14]
TiO_2/ZnO	4−MBA	6.8×10^{5}	10^{-8}	633	[15]
CuO@TiO_2 heterojunction	R6G	/	10^{-8}	532	[16]
Photo-reduced TiO_2@WO_3 nanofibers	R6G	2.45×10^{5}	10^{-8}	633	[17]
Ag@ZnO@Bi_2WO_6	R6G	3.2×10^{5}	10^{-9}	532	[18]
WO_{3-x}/TiO_2	MO	1.2×10^{5}	2.5×10^{-10}	532	This work

References

1. Wang, X.; Shi, W.; Jin, Z.; Huang, W.; Lin, J.; Ma, G.; Li, S.; Guo, L. Remarkable SERS Activity Observed from Amorphous ZnO Nanocages. *Angewandte Chemie International Edition* 2017, *56*, 9851-9855, doi:https://doi.org/10.1002/anie.201705187.

2. Zarei, A.; Shafiekhani, A. Surface-enhanced Raman scattering (SERS) of Methyl Orange on Ag-DLC nanoparticles. *Materials Chemistry and Physics* 2020, *242*, 122559.
3. Wang, P.; Guo, S.; Hu, Z.; Li, T.; Pu, S.; Mao, H.; Cai, H.; Zhu, Z.; Li, H.-Y.; Liu, H. $W_{18}O_{49}$ sensitized with Pd nanoparticles for ultrasensitive ppb-level formaldehyde detection. *Chemical Engineering Journal* 2023, *456*, 140988, doi:https://doi.org/10.1016/j.cej.2022.140988.
4. Zhang, W.; Yuan, T.; Wang, X.; Cheng, Z.; Xu, J. Coal mine gases sensors with dual selectivity at variable temperatures based on a $W_{18}O_{49}$ ultra-fine nanowires/Pd@Au bimetallic nanoparticles composite. *Sensors and Actuators B: Chemical* 2022, *354*, 131004, doi:https://doi.org/10.1016/j.snb.2021.131004.
5. Zhang, A.; Fang, Y. Adsorption orientations and interactions of methyl orange on negatively and positively charged colloidal silver particles. *Journal of Colloid and Interface Science* 2007, *305*, 270-274, doi:https://doi.org/10.1016/j.jcis.2006.09.068.
6. Prakash, O.; Kumar, S.; Singh, P.; Deckert, V.; Chatterjee, S.; Ghosh, A.K.; Singh, R.K. Surface-enhanced Raman scattering characteristics of CuO: Mn/Ag heterojunction probed by methyl orange: effect of Mn^{2+} doping. *J. Raman Spectrosc.* 2016, *47*, 813-818, doi:10.1002/jrs.4904.
7. Yu, T.-H.; Ho, C.-H.; Wu, C.-Y.; Chien, C.-H.; Lin, C.-H.; Lee, S. Metal–organic frameworks: a novel SERS substrate. *Journal of Raman Spectroscopy* 2013, *44*, 1506-1511, doi:https://doi.org/10.1002/jrs.4378.
8. Liu, W.; He, X.; Wang, Z.; Yuan, M.; Zhao, Z.; Ye, X.; Shang, S.; Song, Z.; Huang, L.; Liu, Y.; et al. Geometric and Electronic Structure Modulation to Optimize the Charge Transfer of TiO_2 for Ultrasensitive and Stable SERS Sensing. *Inorganic Chemistry* 2024, *63*, 17608-17616, doi:10.1021/acs.inorgchem.4c02364.
9. Cong, S.; Yuan, Y.; Chen, Z.; Hou, J.; Yang, M.; Su, Y.; Zhang, Y.; Li, L.; Li, Q.; Geng, F.; et al. Noble metal-comparable SERS enhancement from semiconducting metal oxides by making oxygen vacancies. *Nature Communications* 2015, *6*, 7800, doi:10.1038/ncomms8800.
10. Lin, J.; Yu, J.; Akakuru, O.U.; Wang, X.; Yuan, B.; Chen, T.; Guo, L.; Wu, A. Low temperature-boosted high efficiency photo-induced charge transfer for remarkable SERS

activity of ZnO nanosheets. *Chemical Science* 2020, *11*, 9414-9420, doi:10.1039/D0SC02712J.

11. Zheng, Z.; Cong, S.; Gong, W.; Xuan, J.; Li, G.; Lu, W.; Geng, F.; Zhao, Z. Semiconductor SERS enhancement enabled by oxygen incorporation. *Nature Communications* 2017, *8*, 1993, doi:10.1038/s41467-017-02166-z.
12. Zhang, J.; Xing, T.; Zhang, M.; Zhou, Y. Facile preparation of $Cu_{2-x}S$ supernanoparticles with an unambiguous SERS enhancement mechanism. *Chemical Engineering Journal* 2022, *434*, 134457, doi:https://doi.org/10.1016/j.cej.2021.134457.
13. Zhou, L.; Zhou, J.; Lai, W.; Yang, X.; Meng, J.; Su, L.; Gu, C.; Jiang, T.; Pun, E.Y.B.; Shao, L.; et al. Irreversible accumulated SERS behavior of the molecule-linked silver and silver-doped titanium dioxide hybrid system. *Nature Communications* 2020, *11*, 1785, doi:10.1038/s41467-020-15484-6.
14. Wei, J.; Qayum, A.; Jiao, X.; Wang, T.; Chen, D. Photo-reduced WO_3/PAN nanofiber membranes with deposited Ag nanoparticles as efficient SERS substrates. *Applied Surface Science* 2021, *568*, 150936, doi:https://doi.org/10.1016/j.apsusc.2021.150936.
15. Jiang, X.; Xu, L.; Ji, W.; Wang, W.; Du, J.; Yang, L.; Song, W.; Han, X.; Zhao, B. One plus one greater than Two: Ultrasensitive Surface-Enhanced Raman scattering by TiO_2/ZnO heterojunctions based on Electron-Hole separation. *Applied Surface Science* 2022, *584*, 152609, doi:https://doi.org/10.1016/j.apsusc.2022.152609.
16. Yin, W.; An, S.; Cheng, T.; Jiang, L.; Cao, Y. Enhancing SERS sensitivity of semiconductors through constructing CuO@TiO_2 heterojunctions via atomic layer deposition. *Applied Surface Science* 2024, *672*, 160820, doi:https://doi.org/10.1016/j.apsusc.2024.160820.
17. Wei, J.; Yu, K.; Yu, Y.; Li, S.; Yu, H.; Li, B.; Cui, Y.; Abdul, Q.; Chen, Q.; Hao, Z.; et al. Photo-reduced TiO_2@WO_3 electrospun nanofibers for efficient SERS and photoelectrochemical performances. *Composites Communications* 2024, *46*, 101847, doi:https://doi.org/10.1016/j.coco.2024.101847.
18. Korkmaz, I.; Sakir, M.; Sarp, G.; Salem, S.; Torun, I.; Volodkin, D.; Yavuz, E.; Onses, M.S.; Yilmaz, E. Fabrication of superhydrophobic Ag@ZnO@Bi_2WO_6 membrane disc as

flexible and photocatalytic active reusable SERS substrate. *Journal of Molecular Structure* 2021, *1223*, 129258, doi:https://doi.org/10.1016/j.molstruc.2020.129258.
